# Assessments of attrition bias in Cochrane systematic reviews are highly inconsistent and thus hindering trial comparability

**DOI:** 10.1186/s12874-019-0717-9

**Published:** 2019-04-05

**Authors:** Andrija Babic, Ruzica Tokalic, João Amílcar Silva Cunha, Ivana Novak, Jelena Suto, Marin Vidak, Ivana Miosic, Ivana Vuka, Tina Poklepovic Pericic, Livia Puljak

**Affiliations:** 1 Institute of Emergency Medicine in Split-Dalmatia County, Split, Croatia; 2 University of Split School of Medicine, Split, Croatia; 3 Faculty of Medicine, University of Lisbon, Lisbon, Portugal; 4 Center for Evidence-Based Medicine and Health Care, Catholic University of Croatia, Ilica 242, 10000 Zagreb, Croatia

**Keywords:** Systematic review, Cochrane, Attrition bias, Incomplete data, Missing data, Inconsistency

## Abstract

**Background:**

An important part of the systematic review methodology is appraisal of the risk of bias in included studies. Cochrane systematic reviews are considered golden standard regarding systematic review methodology, but Cochrane’s instructions for assessing risk of attrition bias are vague, which may lead to inconsistencies in authors’ assessments. The aim of this study was to analyze consistency of judgments and support for judgments of attrition bias in Cochrane reviews of interventions published in the Cochrane Database of Systematic Reviews (CDSR).

**Methods:**

We analyzed Cochrane reviews published from July 2015 to June 2016 in the CDSR. We extracted data on number of included trials, judgment of attrition risk of bias for each included trial (low, unclear or high) and accompanying support for the judgment (supporting explanation). We also assessed how many Cochrane reviews had different judgments for the same supporting explanations.

**Results:**

In the main analysis we included 10,292 judgments and supporting explanations for attrition bias from 729 Cochrane reviews. We categorized supporting explanations for those judgments into four categories and we found that most of the supporting explanations were unclear. Numerical indicators for percent of attrition, as well as statistics related to attrition were judged very differently. One third of Cochrane review authors had more than one category of supporting explanation; some had up to four different categories. Inconsistencies were found even with the number of judgments, names of risk of bias domains and different judgments for the same supporting explanations in the same Cochrane review.

**Conclusion:**

We found very high inconsistency in methods of appraising risk of attrition bias in recent Cochrane reviews. Systematic review authors need clear guidance about different categories they should assess and judgments for those explanations. Clear instructions about appraising risk of attrition bias will improve reliability of the Cochrane’s risk of bias tool, help authors in making decisions about risk of bias and help in making reliable decisions in healthcare.

**Electronic supplementary material:**

The online version of this article (10.1186/s12874-019-0717-9) contains supplementary material, which is available to authorized users.

## Background

Cochrane systematic reviews are produced using rigorous and evolving methodological standards and are therefore considered the gold standard when it comes to synthesis of evidence. The Cochrane has been at the forefront of applying the methods of evidence-based medicine (EBM) in the treatment and management of various conditions [1].

An important part of the systematic review methodology is appraisal of the risk of bias (RoB) in included studies. The potential effect of bias is that trialists will reach wrong conclusions about efficacy and safety of studied interventions. Bias can, therefore, negatively affect the estimated intervention effects [2].

In Cochrane systematic reviews RoB is appraised using Cochrane RoB tool, which has seven domains; one of them is called ‘incomplete outcome data (attrition bias)’. Incomplete outcome data can yield attrition bias due to amount, nature or handling of incomplete outcome data [3]. The main strength of RCTs is that study arms should generally be balanced in terms of their baseline characteristics, and any imbalance should be result of chance. Attrition can occur if participants are lost to follow-up, or if they miss one or more measurement time points during a trial. Therefore, attrition can lead to bias if the characteristics of participants with missing data are different between the randomized groups [4]. Akl et al. analyzed potential impact of losses to follow-up on the estimates of the effect of treatment in 235 RCTs, and found that different assumptions about outcomes of participants lost to follow-up could change interpretation of results of up to 58% of RCTs published in top medical journals, and a third of the analyzed trials failed to report whether any loss to follow-up occurred [5].

In the Cochrane RoB tool, the authors need to provide judgment about whether this risk is high, unclear or low for each domain. Furthermore, each judgment needs to be accompanied with a supporting explanation called ‘support for judgment’, which “*describes what was reported to have happened in the study, in sufficient detail to support a judgment about the risk of bias”*. The aim of the support for judgment is to ensure transparency about how these judgments about the level of risk of bias were reached [3].

The Cochrane Handbook provides vague instructions about assessing attrition bias, which may lead to inconsistent use of supporting explanations for judgments of attrition bias that one can find in Cochrane reviews. Da Costa et al. have published a study in 2017 about training authors for risk of bias assessment, and showed that “*Kappa values between the minimal training group and reference across items of the risk of bias tool ranged from 0.10 (poor agreement) for incomplete outcome data* (…)” [6]. Therefore, inter-rater agreement in participants with minimal training was worst for the attrition bias domain, compared to other domains of Cochrane RoB. Since Cochrane authors rarely have structured training that was tested in the study of da Costa et al. [6], their data could very well indicate real-world difficulties and discrepancies that authors face when assessing attrition bias.

The aim of this study was to analyze whether Cochrane authors use consistent judgments for different supporting explanations of attrition bias in Cochrane reviews of interventions published in the Cochrane Database of Systematic Reviews (CDSR).

## Methods

### Study design

This was a cross-sectional, primary methodological study in which we analyzed methods of published Cochrane reviews.

### Inclusion and exclusion criteria

Cochrane reviews of interventions published from July 2015 to June 2016 were included by using Advanced search in The Cochrane Library. We excluded diagnostic reviews, empty reviews, overviews of systematic reviews and Cochrane reviews withdrawn in this period and reviews that included only non-randomized studies. If the Cochrane reviews included randomized, quasi-randomized and non-randomized studies, we analyzed attrition bias in the RoB tables for the randomized studies only. Cochrane reviews that had multiple attrition bias judgments assessed for different outcomes in the same study were rare; therefore we reported them separately in order to better describe that methodological approach.

### Screening

Two authors (JASC, LP) independently assessed all titles/abstracts to establish eligibility of Cochrane reviews for inclusion. There were no discrepancies in judgment.

### Data extraction

Data extraction table was developed and piloted using five Cochrane reviews. Seven authors extracted data manually (RT, JASC, IN, JS, MV, IM, IV) and initially another author (AB) checked 10% of the extractions randomly. Discrepancies in data extraction were planned to be resolved by the third author (LP), but we found only several discrepancies, which did not require adjudication by the third author. In 2018, for the purpose of another project we developed customized software acting as a parsing tool, which can extract clearly delimited information from Cochrane reviews. Using the parsing tool, we extracted again the same data for attrition bias from the Cochrane RoB table, and found only 34 discrepancies that needed to be corrected.

The following data were extracted: number of included trials, judgment of attrition risk of bias for each included trial (low, unclear or high) and accompanying ‘support for judgment’. To avoid terminological confusions, instead of ‘support for judgment’ hereby we use the expression ‘supporting explanation’. We also assessed how many Cochrane reviews had inconsistent judgments for the same supporting explanations (i.e. whether they had different judgments for the same supporting explanations). In the main analysis we reported only analysis of attrition bias for included Cochrane reviews with a single judgment (i.e. Cochrane reviews with only one domain for attrition bias, and one judgment in that one domain), regardless of the number of supporting explanations that were provided for that judgment.

In the secondary analysis we investigated i) attrition bias reporting for Cochrane reviews that reported multiple judgments of attrition bias for the same trial (i.e. Cochrane reviews with multiple assessments of attrition bias for the same RCT, where this RoB domain was split into two or more sub-domains analyzing specific aspects of attrition bias), ii) characteristics of risk of bias reporting in Cochrane reviews that did not have attrition bias domain, and iii) characteristics of risk of bias judgment reporting in Cochrane reviews that did not provide judgment in the form of “low, unclear and high”. Specific Cochrane reviews are marked in the body of this manuscript with the serial number of the downloaded record (for example, Cochrane review #1). A list of included and excluded studies with a serial number of each record is in the Additional file 1: Table S1.

### Statistics

Descriptive statistics was performed and data presented as frequencies and percentages. Data were analyzed using Microsoft Excel (Microsoft Inc., Redmond, WA, USA).

## Results

Among 955 Cochrane systematic reviews published from July 2015 to June 2016 we included 729 Cochrane reviews in the main analysis. In the 729 included reviews there were 1–105 included studies (median: 8 studies). In those reviews we found 10,292 attrition bias domains with single judgment about whether the Cochrane review authors found this bias to be low, unclear or high. Although there was a single judgment, 3504/10292 (34%) supporting explanations contained more than one type of explanations related to risk of attrition bias. We categorized these different types of supporting explanations into four categories: #1: percent of attrition in the RCT groups with higher attrition, #2: difference in attrition between the groups, #3: reporting of reasons for attrition and #4: statistical comments. Only 27/10292 (0.26%) of supporting explanations had all four categories of explanations.

### First category: percent of attrition in the RCT groups with higher attrition

In the first category, called ‘percent of attrition in the RCT groups with higher attrition’ a third of supporting explanations were unclear (32%). While there were too many examples of unclear explanations, we provide some examples of explanations categorized by us as unclear explanations in the Table 1. The next most common type of supporting explanations were mentioning only total attrition (16%), indicating there was no attrition (15%) in the trial, providing only number of patients without a percent (11%), or indicating that attrition was not reported in a trial (8.8%) (Table 2).

**Table 1:** Examples of unclear supporting explanations

Study number	Unclear supporting explanation	Judgment
2	All participants were accounted for	Low
12	Outcomes reported for all women randomized	Low
20	Primary outcomes were reported	Low
26	None found	Low
54	Analysed the same number of participants in both groups	Low
66	Expected outcomes reported. Response rates reduced in patients over 4 surveys	Low
80	No study protocol was available	Low
82	It appears that all participants completed the study and contributed data for each outcome at all relevant time points	Low
2	Unclear	Unclear
4	Losses to follow-up were unclear	Unclear
6	It was unclear whether or not there was attrition, or loss to follow-up at final follow-up based on the results section	Unclear
29	No information	Unclear
31	Insufficient information to permit judgment of low risk or high risk	Unclear
32	May be participants randomized who did not complete	Unclear
41	Few data available in conference abstract only	Unclear
66	Unknown	Unclear
442	High attrition (41%)	Unclear
13	Number of drop-outs reported, but no details	High
25	Not all raw data were provided	High
52	Not clear how many withdrew	High

**Table 2:** Number of explanations in a category for percent of attrition per group

Category for percent of attrition per group	N (%)
Unclear	3272 (31.8)
Total attrition only mentioned; attrition per group not reported	1593 (15.5)
No attrition	1544 (15)
Only number of patients, no percent provided	1115 (10.8)
Not reported	901 (8.8)
No explanation for this category	414 (4)
10–20%	359 (3.5)
Above 30%	276 (2.7)
Under 10%	267 (2.6)
20–30%	216 (2.1)
Total attrition reported as percent; attrition per group reported as absolute numbers so it was not possible to judge percent attrition per group	248 (2.4)
Information about attrition provided for one group only	35 (0.3)
‘Support for judgment’ box was blank: no explanation provided for the judgment	27 (0.3)
Above certain percentage that is not precisely defined	13 (0.1)
Under certain percentage that is not 10%	6 (0.06)
There was no supporting explanation because RoB table did not have a domain for attrition bias at all	6 (0.06)
Total	10,292 (100)

We categorized reported percent of attrition in the group with higher attrition into four categories: attrition under 10%, between 10 and 20%, between 21 and 30% and above 30%. Since some Cochrane reviews had multiple supporting explanations for a single judgment, we analyzed separately only reviews where the only supporting explanation was about percent of attrition in the study groups (Table 3). The purpose of this analysis was to see whether Cochrane authors use consistent judgments for various thresholds of attrition in this category of supporting explanations. In the Table 3 we listed total number of Cochrane reviews that had supporting explanations related to percent of attrition in the RCT groups with higher attrition. However, on the right side of the Table 3 we presented data only for reviews where the only supporting explanation was about percent of attrition in the study group because only for these Cochrane reviews we can be sure that the single judgment applies only to that comment. As Table 3 indicates, Cochrane authors use very heterogeneous judgments for each category of comment.

**Table 3:** Frequency of different judgments for the same supporting explanation related to percent of attrition in RCT groups and comments about statistics

Supporting explanation *n* = total number of Cochrane reviews that had this supporting explanation *N* = number of analyzed Cochrane reviews	Risk of bias judgment		
	Low, N (%)	Unclear, N (%)	High, N (%)
*Percent of attrition in the RCT groups with higher attrition*			
Attrition between the groups was under 10%, *n* = 264, *N* = 122	101 (82.8)	16 (13.1)	5 (4.1)
Attrition between the groups that was 10–20%, *n* = 354, *N* = 143	91 (63.6)	28 (19.6)	24 (16.8)
Attrition between the groups that was 21–30%, *n* = 215, *N* = 60	34 (56.7)	5 (8.3)	21 (35)
Attrition between the groups that was above 30%, *n* = 276, *N* = 70	18 (25.7)	9 (12.9)	43 (61.4)
*Supporting explanations about statistics*			
ITT analysis used, *n* = 825, *N* = 193	140 (72.5)	21 (10.9)	32 (16.6)
ITT analysis was not used, *n* = 238, N = 35	20 (57.1)	9 (25.7)	6 (17.1)
PP analysis used, *n* = 81, N = 8	7 (87.5)	1 (12.5)	0 (0)
LOCF analysis used, *n* = 66, *N* = 25	13 (52)	3 (12)	9 (36)

*Abbreviations: ITT* intention-to-treat, *LOCF* last observation carried forward, *PP* per protocol, *RCT* randomized controlled trial,

### Second category: difference in attrition between the groups

In the second category of supporting explanations about difference in attrition between the groups, 302/10292 (2.9%) explanations reported this category, and in all of them it was reported if the difference was above 10%.

### Third category: reporting of reasons for attrition

There were 2157/10292 (21%) supporting explanations related to reasons for attrition. The majority of these explanations referred to reasons for attrition that were reported in a trial, while the remaining supporting explanations indicated either that reasons for attrition were not reported in a trial, or that they were inadequately reported (Table 4).

**Table 4:** Number of supporting explanations in a category for reporting reasons for attrition

Category: reporting of reasons for attrition	N (%)
Reasons reported	1697 (16.5)
Reasons not reported	370 (3.6)
Inadequately reported	90 (0.9)
Total	2157 (21)

### Fourth category: supporting explanations about statistics

We found 1572/10292 (15.3%) supporting explanations related to statistics; Table 5 lists all of them in a way that they were described by the Cochrane review authors themselves. Most of the explanations about statistics were referring to presence or absence of intention-to treat analysis (ITT), per protocol analysis (PP) or last observation carried forward (LOCF) (Table 5). Detailed analysis of risk of bias judgment categories was shown only for the most commonly used categories that reported only supporting explanation about statistics; for each statistical comment, Cochrane authors had highly heterogeneous judgments regarding their impact on risk of attrition bias (Table 3).

**Table 5:** Supporting explanations about statistics used that was related to attrition bias

Statistical information	N (%)
ITT	826 (8)
No ITT	238 (2.3)
PP	88 (0.9)
ITT, LOCF	87 (0.8)
LOCF	67 (0.7)
ITT not reported	47 (0.5)
ITT, PP	34 (0.3)
Completer analysis	27 (0.2)
mITT	25 (0.2)
Sensitivity analysis	15 (0.1)
BOCF	12 (0.1)
ITT, BOCF	8 (0.08)
Analysis not described	6 (0.06)
Available case analysis	5 (0.05)
ITT, Completer analysis	5 (0.05)
LOCF, BOCF	5 (0.05)
ITT analysis may have been of value	4 (0.04)
ITT, PP, LOCF	4 (0.04)
ITT, LOCF, WOCF	4 (0.04)
LOCF, PP	4 (0.04)
Partial ITT	4 (0.04)
WOCF	3 (0.03)
Unclear whether LOCF was used	3 (0.03)
ITT inadequate	3 (0.03)
Some participants were excluded from analysis	3 (0.03)
No ITT, PP	3 (0.03)
BOCF, WOCF	2 (0.02)
ITT, LOCF, NRI	2 (0.02)
No LOCF	2 (0.02)
We have not been able to re-analyse the outcomes for all of the enrolled infants (ITT)	1 (0.01)
LOCF, Sensitivity analysis	1 (0.01)
ITT, PP, LOCF, Sensitivity analysis	1 (0.01)
The trial states that the analysis was performed on an ITT basis, but the data seems to have been analysed on-treatment	1 (0.01)
ITT analysis possible	1 (0.01)
ITT analysis conducted but unclear how missing data were dealt with	1 (0.01)
PP, FAS	1 (0.01)
It is likely that the principle of ITT analysis was violated	1 (0.01)
Statistical analysis used the APT	1 (0.01)
Missing outcome data imputed in analysis	1 (0.01)
True ITT analysis was difficult	1 (0.01)
Missing participants were omitted from the analysis	1 (0.01)
Although the study was set up to be analysed on ITT basis, the participants with missing outcomes were not included in the primary analysis	1 (0.01)
ITT done only for *P* value	1 (0.01)
Not strict ITT analysis	1 (0.01)
mITT, but unclear how missing data were dealt with	1 (0.01)
ITT, WOCF	1 (0.01)
mITT, LOCF	1 (0.01)
mITT, PP	1 (0.01)
Equal distribution among groups, ITT analysis not necessary	1 (0.01)
It was unclear if data analysis was PP or ITT	1 (0.01)
The results are presented as available case analysis rather than ITT. The authors present a sensitivity analysis	1 (0.01)
No information about whether an ITT analysis was undertaken and, if so, how missing data were imputed	1 (0.01)
This is an “as treated” as opposed to an ITT analysis	1 (0.01)
LOCF, BOCF, SOCF	1 (0.01)
ITT, PP, mITT	1 (0.01)
ITT, No sensitivity analysis	1 (0.01)
LOCF, Completer analysis	1 (0.01)
Large number of cross-overs made ITT impossible after the first phase	1 (0.01)
Unclear if ITT	1 (0.01)
ITT, PP, Sensitivity analysis	1 (0.01)
No ITT, Completer analysis	1 (0.01)
No mention of how missing data from participants who dropped out were dealt with, e.g. ITT analysis	1 (0.01)
ITT, Sensitivity analysis	1 (0.01)
No sensitivity analysis	1 (0.01)
LOCF, WOCF	1 (0.01)

Abbreviations: *ITT* intention-to-treat analysis, *PP* per protocol analysis, *LOCF* last observation carried forward, *mITT* modified intention-to-treat analysis, *BOCF* baseline observation carried forward, *WOCF* worst observation carried forward, *NRI* non-responder imputation, *FAS* full analysis set, *APT* all patients treated, *SOCF* screening observation carried forward

There were 35 Cochrane reviews that indicated that it was unclear whether ITT analysis was used or not, because its usage was not described. We did not analyze this group of CRSs because none of those listed this item as the only supporting explanation for risk of attrition bias judgment.

### Inconsistencies in judgments in single Cochrane reviews

We found only 34/729 (4.7%) Cochrane reviews that had inconsistencies in judging risk of attrition bias in the same review. This means that they gave different judgment for the same explanation. For example, “No incomplete outcome data” was judged as either low or unclear risk of bias in the review #210. In the review #255 explanation “No pre-publication protocol identified” was judged either as unclear or high. In the review #277 “No missing data” was judged as low or unclear. In the review #330 “No withdrawals mentioned” was judged as either low or unclear risk of attrition bias. There were 66/729 (9.1%) Cochrane reviews for which this analysis was not applicable because they included only one trial. All the other reviews had consistent judgments for the given supporting explanations.

### Secondary analysis: studies with multiple judgments of attrition bias for the same study

We found 27 Cochrane reviews that had multiple assessments of attrition bias for the same RCT. They had 2–7 multiple assessments separately, which we categorized in assessments related to aspects of attrition bias, time, objectivity and clinical outcomes.

Five Cochrane reviews had separate assessments of different aspects of attrition bias were assessments of drop-outs, participants analyzed in the group to which they were allocated and whether ITT analysis was performed. Seven reviews had assessments related to time were multiple assessments for short-term or long-term outcomes, sometimes defined with specific time-frame (i.e. before or after 12 weeks or childhood outcomes), or end-of-intervention and end of follow-up. Five Cochrane reviews had separate assessments for subjective and objective outcomes. One of them specified what was a subjective and what an objective outcome was. Ten reviews had separate assessments for different clinical outcomes (Table 6). The review authors did not analyze all these sub-domains for all studies included in those reviews.

**Table 6:** Description of domains in Cochrane reviews that had multiple separate domains for assessing attrition bias for different outcomes

Study number	Names of separate domains for attrition bias in the Risk of Bias table
158, 197	Short-term, long-term
240	End-of-intervention, end of follow-up
250, 459, 533	Subjective outcome measures, objective outcome measures
285	Clinical heart failure, subclinical heart failure (dichotomous and/or continuous), overall survival, tumor response, quality of life, adverse effects, adverse effects other than cardiac damage
302	Drop-out rate described and acceptable, participants analyzed in the group to which they were allocated
312	Mortality (all cause), hospital readmissions (all cause), hospital readmissions (due to adverse drug events), hospital emergency department contacts (all-cause), hospital emergency department contacts (due to adverse drug events), adverse drug events
316	Adverse events: hypothyroidism, development or worsening of Graves’ ophthalmopathy, health-related quality of life, participants in euthyroid state, recurrence of hyperthyroidism, socioeconomic effects
324	12 weeks or less, after 12 weeks
340	Primary outcomes, secondary outcomes
346	All outcomes: drop-outs, all outcomes: ITT analysis
394	Time to resolution of diabetic ketoacidosis, all-cause mortality, hypoglycemic episodes, morbidity, socioeconomic effects
427	Drop-outs reported, ITT analysis reported
499	Objective outcome (deaths), subjective outcome (quality of life)
638, 795	Drop-outs, ITT analysis
641	Pain, function
722	Short term follow-up (up to 3 months), longer term follow-up
761	Consumption outcome, selection outcome
805	Hemodynamic data, clinical outcomes
867	Survival, tumor response, toxicity, quality of life
943	Short-term outcomes, childhood outcomes
946	All outcomes, ITT analysis
949	Wound healed, wound area, time to healing
951	Pain, swelling, function, adverse effects

### Cochrane reviews that did not have a domain for attrition bias in the RoB table

There were 12 Cochrane reviews that did not have a domain for attrition bias at all in the RoB table. They were not included in the main analysis, and hereby we report characteristics of their RoB tables. Five reviews analyzed only 1 RoB domain, and this was ‘Allocation concealment in four cases (reviews #341, #465, #672 and #904) and ‘Method for selecting cases to adjudicate?’ in one case (reviews #269). One review analyzed 3 RoB domains (Random sequence generation, Allocation concealment and Blinding as one domain for all outcomes), but not attrition bias (review #294). Three reviews analyzed 4 RoB domains; one of them analyzed ‘Random sequence generation’, ‘Allocation concealment’, ‘Blinding of outcome assessment’, ‘Selective reporting’ (review #585) and two analyzed domains for ‘Random sequence generation’, ‘Allocation concealment’, ‘Blinding of participants and personnel (performance bias)’, ‘Size’ (review #924, #936). Two Cochrane reviews analyzed five RoB domains (review #174, #947) and one analyzed six RoB domains – but none of the domains were attrition bias (review #309).

### Risk of bias assessed with ‘yes’ or ‘no’ judgments

In 4/729 Cochrane reviews (0.5%) there was no standard judgment of risk of bias as high, unclear or low; instead RoB was judged as yes, unclear, no, or yes/no (reviews #212, #292, #830 and #884). In one review risk of bias was graded as “low, unclear or high”, but in the supporting explanation also rated as A – Adequate, B – Unclear, C – Inadequate (review #244).

### Other inconsistencies that were encountered

Several Cochrane reviews had different name of the relevant domain. In the review #641 the domain was called “Intention-to-treat analysis performed?”, in the #419 “Losses to follow-up taken into account?” and in the review #873 “Complete follow-up?”

### Explanations that should not be used for judging attrition bias

Finally, we decided to report examples of curious explanations for attrition bias judgments in Table 7. It appears to us that such explanations should not be used for explaining risk of attrition bias judgments.

**Table 7:** Examples of curious supporting explanations for attrition bias judgments that may not appear to be suitable for judging this risk of bias domain

Study number	Support for judgment	Judgment for risk of attrition bias
82	Chinese article - unable to ascertain	Unclear
144	This study was a feasibility study. Only 1 woman received the intervention. This study contributed no data to the review.	Unclear
255	No pre-published protocol identified	High or unclear
256	If we assume a person works for 40 h per week, then for 28 participants the working hours will be 8960 h for 8 weeks (4 weeks intervention and 4 weeks control period). However the study reported only 7729 working hours based on accelerometer data	High
376	This is not clear from the paper. Author contacted, but when he moved jobs, the data files for this study were deleted	Unclear
490	137 minus 28 equals 109, not 108	Unclear
492	Exact time periods of ‘before and after’ accident data were unclear. Authors reported that they “should be 3 to 5 years”.	Unclear
494	1 - A reasonable account of how attrition was dealt with is given, but no specific reference to CONSORT	Low
517	Documented evidence that the CONSORT guidelines have been followed	Low
606	Data sparse largely narrative style	Unclear
699	Numbers do not always add up - query if N for outcomes are based on those who answered specific questions on follow-up?	High
727	Data of drop-outs was censored.	Low
730	Eleven patients were withdrawn before random assignment: 1 declined further participation, 8 were withdrawn by their physician, and 2 did not meet the entry criteria	Low
744	Publication is in German and our translation is incomplete.	Unclear
835	Differences in baseline characteristics of questionnaire responders vs non-responders (western ethnicity in 81% vs 54%, mean age 31 vs 28 years, median blood loss 1500 vs 1150 mL). Big difference in compliance to allocated treatment: 8 vs 34. The design of this trial carries a high risk for selecting the study population	High
838	Primary and secondary endpoints not specified directly but do address aims	Low
849	“The situations to consider eliminating the subject from data analysis did not arise”	Low
850	No Table 1 to clearly describe participant characteristics.	High
854	Duration of study not defined	High
854	Criteria for kidney disease not defined	Unclear
873	Denominators inconsistent in study	Unclear

## Discussion

We found high inconsistency in the assessment of risk of bias related to incomplete outcome data, i.e. attrition bias in Cochrane systematic reviews. Cochrane authors do not have uniform approach to judging attrition bias. We did not observe clear numerical rules about the percent of attrition in trial groups or clear rules about statistics that was used or not used, that were consistently labeled as low, unclear or high risk of bias. One third of Cochrane review authors had more than one category of explanations; some had up to four different categories. Inconsistencies were found even with the number of judgments, names of risk of bias domains and different judgments for the same explanations in the same review.

Cochrane Handbook indicates that “*Missing outcome data, due to attrition (drop-out) during the study or exclusions from the analysis, raise the possibility that the observed effect estimate is biased.*” The term attrition bias is used for both exclusions and attrition [3]. Besides numerical indicators of attrition – absolute numbers and frequencies – that provide information about the magnitude of attrition, in the context of this domain of risk of bias different statistical methods for imputing missing data are often mentioned. For example, trial authors can use ITT analysis, or a ‘modified ITT analysis’. However, it has been reported that the term ‘ITT analysis’ does not always have a clear and consistent definition, and that it is not consistently used in trial reports [7]. The same was concluded for the modified ITT analysis and therefore it has been recommended by the Cochrane Handbook that the review authors should always ask information about who exactly was included in such analysis [3]. Simple imputations, such as last observation carried forward (LOCF) remain very popular despite warnings of statisticians against their use [8].

Judgments about different statistical methods varied in our analysis; we found very inconsistent judgments for different statistical methods. If we want to judge by the frequency of statistical comments in reviews where this was the only available explanation, we could not reach any conclusion, because the majority of authors judged presence of ITT analysis with low risk of bias, but also in the group that reported explicitly that there was no ITT analysis, this absence of ITT analysis was also predominantly judged with low risk of bias. Using *per protocol* analysis was mostly judged as low risk of bias, as well as LOCF analysis.

It has been published previously that attrition under 5% is not likely to introduce bias, while attrition rates above 20% raise concerns about the study validity [9]. While Cochrane handbook does not give clear guidance about the total attrition or attrition per group regarding specific numerical values, there is an example in the Fig. 8.6.a. in that handbook: “*17/110 missing from intervention group (9 due to ‘lack of efficacy’); 7/113 missing from control group (2 due to ‘lack of efficacy’)”* that is judged as high risk [3]; in this example the first group has attrition of 15%. If a Cochrane author should follow this example, then attrition that is 15% or above per group should be labeled as high risk of bias. In Table 8, we present examples of vague instructions for Cochrane authors regarding judgments of attrition bias, in line with the current instructions for judging attrition bias that are available in the Cochrane Handbook in Table 8.5.d., which gives authors instructions about specific situations where each domain should be judged as low, unclear or high [3].

**Table 8:** Examples of vague instructions for Cochrane authors regarding judgments of attrition bias

Quote from a Cochrane review	Comment of authors of this study
Missing outcome data balanced in numbers across intervention groups, with similar reasons for missing data across groups	There is no quantitative measure of “balanced”
For dichotomous outcome data, the proportion of missing outcomes compared with observed event risk not enough to have a clinically relevant impact on the intervention effect estimate	There is no quantitative measure of “not enough”
Missing data have been imputed using appropriate methods	Not specified what is considered by Cochrane to be “appropriate methods”
Reason for missing outcome data likely to be related to true outcome, with either imbalance in numbers or reasons for missing data across intervention groups	There is no quantitative measure of “imbalance”
Potentially inappropriate application of simple imputation.	Not specified what is considered to be “inappropriate application”

In our study we found that numerical indicators for what represents attrition were widely inconsistent. When we categorized reported percent of attrition in the group with higher attrition and which threshold was predominantly judged in a certain way, attrition in a group that was under 10% was judged as low risk of bias in 83% of the cases, attrition 10–20% was judged as low risk of bias in 64% of cases, attrition 20–30% was judged as low risk of bias in 57% of cases. If we judge from the majority opinion of Cochrane authors, threshold of ‘above 30% is considered predominantly high risk of bias because 61% of judgments indicated so in Cochrane reviews where this was the only judgment so we could isolate the effect of this category for the overall judgment.

As for the risk of bias as a tool, it has been reported that it has low reliability between individual reviewers and across consensus assessments of reviewer pairs [10]. It has been argued that low reliability of the RoB assessment can have negative effects on decision making and quality of health care [11]. It has also been shown by da Costa et al. that standardized intensive training on RoB assessment may significantly improve the reliability of the Cochrane RoB tool [6]. However, our study points out that we would need first to have standardized instructions about what situations really represent risk of attrition bias. Having clear instructions, such as “attrition above 20% represents high risk of attrition bias” it would be much easier to achieve higher reliability of RoB assessment, even without formal training.

Instructions for assessing risk of attrition bias should include specific indications about all categories of assessment that should be appraised. It should be clearly specified which of those categories systematic review authors should assess, such as four that we used in this manuscript, including percent of attrition per group and difference between the groups, whether reasons for attrition were reported or not, and what is the appropriate statistics for dealing with attrition. If the authors do not have clear guidance about assessment of attrition bias, they can behave as we found – they can use one or more of those categories for their attrition RoB assessment as they personally see fit.

Some authors used multiple judgments for different follow-ups or different outcomes. This also introduces inconsistency in the attrition RoB assessment. Just as the option for authors to change the titles of attrition RoB domains in the RoB table in a Cochrane review.

In our previous analyses of other domains of Cochrane RoB tool in Cochrane reviews have shown that judgments and supports for judgments in those other domains were very inconsistent as well [12–14], further supporting the idea that more attention needs to be devoted to the way authors use this tool.

New version of Cochrane RoB tool, called RoB tool 2.0 is being developed, and its draft version is available online [15]. The draft version of the RoB tool 2.0 has five domains, the domain comparable to the current “Incomplete outcome data (attrition bias)” is the third out of five domains, called “Bias due to missing outcome data”. The RoB tool 2.0 has signaling questions in each domain, and this particular domain has three signaling questions [15]. Theoretically, having three signaling questions could help authors to produce three categories of responses, but this will not be the case because some of those signaling questions address more than one category of attrition bias, in the context of categories defined in this manuscript. For example, elaboration for the second signaling question includes both discrepancies in missing data across intervention groups, and reporting reasons for missing data [15].

Furthermore, we consider that this specific domain in the RoB tool 2.0 is not even a step forward in terms of specific instructions to Cochrane authors, because the field “elaboration” of the signaling questions is still as vague as in the current RoB tool, and could be interpreted by Cochrane authors in various ways. The first signaling question is *“3.1 Were outcome data available for all, or nearly all, participants randomized?”*. In the elaboration for the first signaling question there is a phrase *“low or modest amount of missing data”*, but it is not specified what exactly should Cochrane authors consider as “low” and “modest”. The elaboration further says *“availability of data from 95% (or possibly 90%) of the participants would often be sufficient*”, but it is unclear what is "often" and when is this not sufficient [15].

The second signaling question is *“Are the proportions of missing outcome data and reasons for missing outcome data similar across intervention groups?”* Elaboration does not give specific instructions about the magnitude of discrepancies; instead it says “*minor degree of discrepancy*” [15].

The third signaling question is “*Is there evidence that results were robust to the presence of missing outcome data?”*, and the elaboration says *“Evidence for robustness may come from how missing data were handled in the analysis and whether sensitivity analyses were performed by the trial investigators, or from additional analyses performed by the systematic reviewers”*. [15]. Again, to us, this elaboration does not give specific instructions to Cochrane authors, and may result in heterogeneous perception and judgment.

Future studies on this topic should explore how to reduce inconsistency in assessment of attrition RoB, and they should attempt to reach consensus about what exactly should be assessed in this RoB domain.

## Conclusion

We found very high inconsistency in methods of appraising risk of attrition bias in recent Cochrane reviews. Systematic review authors need clear guidance about different categories they should assess and judgments for those explanations. Clear instructions about appraising risk of attrition bias will improve reliability of the Cochrane risk of bias tool, help authors in making decisions about risk of bias and help in making reliable decisions in healthcare.

## Additional file

Additional file 1: **Table S1.** A list of included and excluded studies with a serial number of each record. The supplementary table contains a full list of included and excluded studies. Data are arranged in four columns. The first column contains a serial number of each study; second column contains title of a review; third column a remark about whether the review was included in the study or not (yes or no); fourth column describes reason for exclusion if a review was not included. (XLSX 65 kb)

## Abbreviations

APT: All patients treated
BOCF: Baseline observation carried forward
CDSR: Cochrane Database of Systematic Reviews
EBM: Evidence-based medicine
FAS: Full analysis set
ITT: Intention-to treat analysis
LOCF: Last observation carried forward analysis
mITT: Modified intention-to-treat analysis
NRI: Non-responder imputation
PP: Per protocol analysis
RCT: Randomized controlled trial
RCTs: Randomized controlled trials
RoB: Risk of bias
SOCF: Screening observation carried forward
WOCF: Worst observation carried forward
